# Complex Karyotype in Hematological Diseases: A 6-Year Single Centre Study from Pakistan

**DOI:** 10.1155/2018/2019239

**Published:** 2018-06-03

**Authors:** Samra Waheed, Jawad Hassan, Maliha Naz, Sidra Maqsood, Madiha Abid, Saira Shan, Muhammad Nadeem, Tahir S. Shamsi

**Affiliations:** National Institute of Blood Disease and Bone Marrow Transplantation, Karachi, Pakistan

## Abstract

**Background:**

Most of the hematological disorders are heterogenous with regard to morphology, immunophenotype, and genetic rearrangements. Multiple recurrent chromosomal aberrations have been identified by conventional cytogenetic analysis, which is now widely recognized as one of the most important diagnostic and prognostic determinants in these patients. Though rarer, complex karyotype has been associated with worst prognosis.

**Materials and Methods:**

A total of 1185 bone marrow or peripheral blood cytogenetics samples were taken with different hematological diseases. They included both benign and malignant disease entities. In each case, cells were cultured and conventional cytogenetic analysis was performed.

**Results:**

Among 1185 subjects, 41 (3.4%) patients possessed complex cytogenetic abnormalities. Out of these 41, 33 (80%) were males. The mean age was 37 years (median age 39 years). Myelodysplastic syndromes had the most numbers of complex karyotypes (8%), followed by acute myeloid leukemia (7%) and acute lymphoblastic leukemia (4%). Also we found few patients with acute promyelocytic leukemia, aplastic anemia , chronic myeloid leukemia, and diffuse large B cell Lymphoma possessing complex karyotype. Frequencies of different cytogenetic abnormalities were assessed with respect to disease as well as independently. Trisomy 21 was the most common chromosomal abnormality found in 28% of patients.

**Conclusion:**

Complex karyotype was most frequently associated with myelodysplastic syndromes and acute myeloid leukemia. Trisomy 21 and deletion 5q were the commonest cytogenetic abnormalities found. We also assessed complex karyotype in benign diseases and detected one patient of aplastic anemia with complex karyotype. This is the first study highlighting the presence of complex karyotypes in hematological disorders in our region.

## 1. Introduction

Cytogenetic analysis of hematological disease is an important methodology used by clinicians and researchers. Observations have shown that clonal chromosomal abnormalities possess both diagnostic and prognostic significance. Either bone marrow or peripheral blood cells may be used to prepare chromosome spreads for cytogenetic analysis. The term complex/aberrant is designated to describe karyotypes with multiple unrelated cytogenetic abnormalities. Technically, any karyotype with at least 3 chromosome aberrations, regardless of their type and the individual chromosomes involved, can be referred to as “complex karyotype” [1, 2]. Complex cytogenetics is associated with potentially adverse outcomes and higher relapse rates with conventional treatment options [3, 4]. The number and complexity of cytogenetic abnormalities that occur in hematological malignancies and the multiple ways in which each can affect patient's care and counseling make the evaluation and interpretation of cytogenetic abnormalities a challenging task. Cytogenetics is the most important prognostic factor for predicting remission rate, relapse, and overall survival in most of the hematological malignancies [5, 6]. However, the importance of cytogenetic analysis in nonmalignant diseases is still uncertain; they either transform to malignancy at some point or remain benign.

The aim of this study was to calculate the frequency of complex cytogenetic abnormalities in malignant and nonmalignant hematological disease in 6-year period at a single institution. As complex karyotype is associated with the worst prognosis, the survival rates were not evaluated.

## 2. Materials and Methods

This study is a descriptive, retrospective analysis done at the National Institute of Blood Disease and Bone Marrow Transplantation from January 2012 to July 2017. We evaluated the diagnostic cytogenetic analysis reports of 1185 patients. Patients of all ages, both genders, with diagnosed or undiagnosed suspected hematological diseases were included. For the diagnosis of all malignant disorders, WHO lymphoid and myeloid neoplasms guidelines were followed and for aplastic anemia, Camitta's classification was followed. All samples of cytogenetics analysis were collected, processed, and analyzed at the National Institute of Blood Disease and Bone Marrow Transplantation; however, many samples were advised by physicians outside the hospital. Written informed consent was taken at the time of the procedure from each patient. Peripheral blood or bone marrow samples were collected. Chromosome analysis required five principal steps: (1) cell culture, (2) harvest of metaphase chromosomes, (3) chromosome preparation, (4) banding and staining using giemsa and trypsin, and (5) analysis by light microscopy or karyotype assisted computer analysis [7]. The addition of colchicine (or colcemid) pretreatment results in mitotic arrest and that treatment of arrested cells with a hypotonic solution like potassium chloride improved the yield and quality of metaphases spreads.

## 3. Statistical Analysis

SPSS software (version 23) was used to calculate the frequency of qualitative variables, i.e., gender, complex karyotype, and distribution of complex karyotype with respect to hematological diseases. Mean and standard deviation of quantitative variables such as age were also measured.

## 4. Results

A total of 1185 patients were analyzed for cytogenetic analysis from January 2012 to June 2017. Complex cytogenetic was found in 41 patients (3.4%). Out of these 41, 33 (80%) were males (Table 1). The mean age of patients was 37 years. We assessed all hematological diseases and subcategorized the patients with respect to diagnosis.

**Figure 1:**
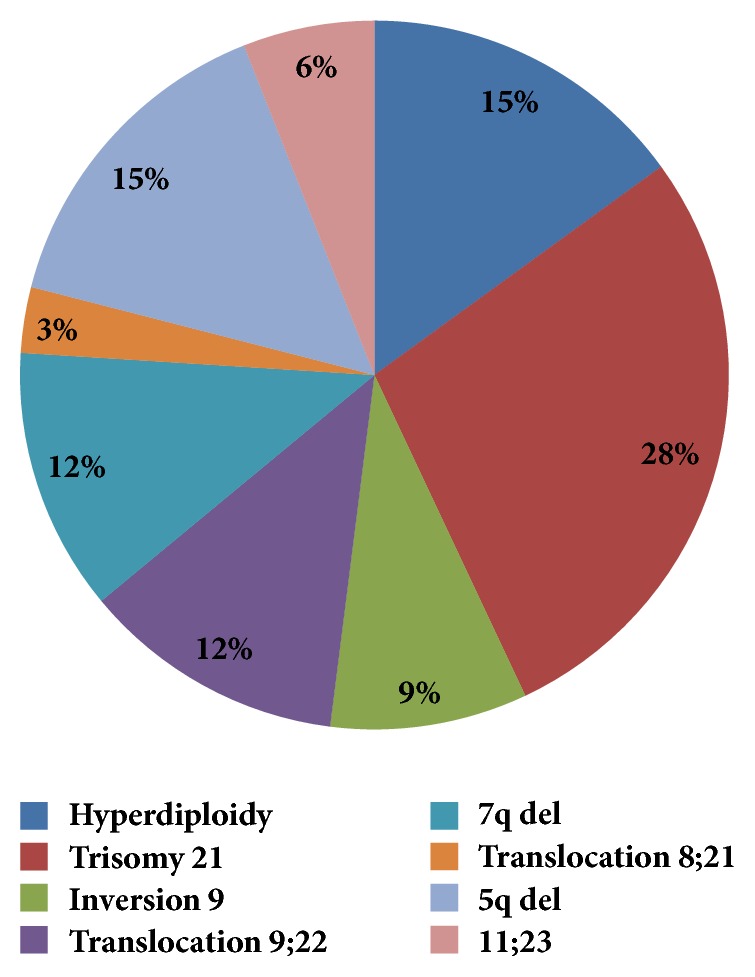
Frequencies of chromosomal abnormalities in complex karyotypes (N=41).

The most common hematological entities possessing complex cytogenetics were myelodysplastic syndromes and acute myeloid leukemia. Other diseases with complex karyotype include acute lymphoblastic leukemia, acute promyelocytic leukemia, chronic myeloid leukemia, diffuse large B cell lymphoma, and aplastic anemia diagnosed on bone marrow biopsy (Table 2). We also assessed presence of different cytogenetic abnormalities commonly reported in complex karyotype. Trisomy 21 was most common cytogenetic abnormality presented in 28% of all complex karyotypes followed by hyperploidy that was 15% (Figure 1).

These karyotypes were also quantified with respect to hematological diseases. In myelodysplastic syndromes deletion 5q was most commonly present; 4 patients (67%) had this deletion along with other abnormal karyotypes. In acute myeloid leukemia and acute lymphoblastic leukemia, trisomy 21 was most common karyotype occurring in 29% and 60% of patients, respectively (Table 3).

## 5. Discussion

For the past three decades, cytogenetic studies of hematological disorders indicate that each and every case is equally and critically important [8, 9]. There are increasing numbers of balanced rearrangements associated with distinct cases and clinical features, suggesting that chromosomal abnormalities reflect basic differences in leukemia biology [10, 11]. Furthermore, clonal cytogenetic abnormalities are one of the most important factors in predicting clinical outcomes in leukemia and are used to guide risk-adapted treatment strategies [12]. In our study, we assessed the frequency of complex karyotypes in hematological diseases and manifestations. Though the occurrence of complex karyotype is rarer, its significance cannot be overruled. The mean age in our study was 37 years, which is more or less the same as that in the other Asian studies [6]. However, the mean age is above 50 in the studies of European origin [13]. This difference may be because of the increased life expectancy in the latter countries. Majority of the patients in our study were males, which is in accordance with previous studies [14, 15]. Myelodysplastic syndromes and acute myeloid leukemia had the highest number of patients possessing complex cytogenetics, which is similar to the results of previous studies done internationally [16–18]. As a well-known fact, acute myeloid leukemia and myelodysplastic syndrome both have poorer prognosis and presence of complex karyotype is one of the many factors involved. Trisomy 21 was the most common karyotype found in our study, followed by hyperploidy and deletion 5q. All these results correlate with the international data [19, 20](Cortes JE et al., 1995; Xiao Fe Yang et al., 2012). Also we acknowledged that trisomy 21 was a common finding in majority of acute myeloid leukemia and acute lymphoblastic leukemia patients with complex karyotype. Though no local data on complex karyotype is available, international studies have shown similar results[10, 21]. In myelodysplastic syndromes, deletion 5q was the most common karyotype abnormality associated with complex karyotype in our study. Various studies from Europe have shown the presence of deletion 5q with complex cytogenetics being associated with worst outcome [22, 23]. All the patients with acute myeloid leukemia, acute lymphoblastic leukemia, and myelodysplastic syndromes died in our study due to either primary disease, infections, or treatment related adverse events. In our study, we have reported two patients of acute promyelocytic leukemia having complex karyotype with translocation (15;17). Few studies have reported acute promyelocytic leukemia with complex karyotype and worst prognosis, refractory disease, and increased relapse rate [24]. However, in our study both the patients are disease-free and off-therapy till date. Two patients in this study were diagnosed as chronic myeloid leukemia, having translocation (9;22) along with complex karyotype. One of the patients died due to blastic transformation while on treatment. The other patient is alive till date but not in molecular or cytogenetic remission, though maintaining the peripheral counts. This patient was never compliant with the medicine. To the best of our knowledge, few case reports have been published internationally reporting chronic myeloid leukemia with complex karyotype and molecular remission [25]. One of our patients diagnosed as aplastic anemia on bone marrow biopsy had complex karyotype and died due to septic shock early during the disease course. International studies have revealed cases of aplastic anemia with complex karyotype, either transforming to acute leukemia or dying earlier due to infections [26]. One of the patient, diagnosed as diffuse large B cell lymphoma on bone marrow and lymph node biopsy, received chemotherapy but was lost to follow up. Though uncommon, diffuse large B cell lymphoma has been reported with complex karyotype, with poorer prognosis[27]. The frequency of complex karyotypes in different diseases mentioned in our study is similar to other international studies. Limitations of our study were small sample size and therefore we could not comment on overall survival, treatment-free survival, and worst prognosis already associated with complex karyotype. To the extent of our knowledge, no study has yet been published comprising solely complex karyotypes and their association in all the hematological diseases either benign or malignant.

## 6. Conclusion

A complex cytogenetic abnormality encompasses importance in the diagnosis and prognosis of various hematological disorders. Its frequency in different hematological conditions implies its importance. In our study trisomy 21 and deletion 5q were the commonest cytogenetic abnormalities found. Moreover complex karyotype was most frequently associated with myelodysplastic syndromes and acute myeloid leukemia. We also assessed complex karyotype in benign diseases, though not significant; one patient with diagnosis of aplastic anemia had the complex cytogenetics. This is the first study highlighting the presence of complex karyotypes in hematological disorders in our region, and to our knowledge, no study has yet been published comprising solely complex karyotypes.

## Figures and Tables

**Table 1:** Gender-wise distribution of complex karyotypes (N=41).

	**No. of patients** **(N)**	**Percentage** (%)
**Male**	33	80

**Female**	8	20

**Table 2:** Frequency of complex karyotypes with respect to hematological diseases (N=1185).

**Disease**	**Total Karyotypes**	**Complex Karyotypes**	**Percentage **(%)
**AML**	249	18	7

**All**	234	9	4

**MDS**	95	8	8

**Acute promyelocytic leukemia**	50	2	4

**CML**	175	2	1

**AA**	55	1	2

**LPD**	42	1	2

**Others**	285	0	0

**Table 3:** Distribution of chromosomal abnormalities in complex karyotype with respect to hematological diseases (N=41).

	**AML** **N **(%)	**All** **N **(%)	**MDS** **N **(%)	**Acute promyelocytic leukemia** **N **(%)	**Others** **N **(%)
**Hyperdiploidy**	2 (12)	1(20)	-	1(100)	1(25)

**Trisomy 21**	5(29)	3(60)	-	-	1(25)

**Inversion 9**	2(12)	1(20)	-	-	-

**Translocation 9;22**	3(17)	-	-	-	1(25)

**7q del**	1(6)	-	2(33)	-	1(25)

**Translocation 8;21**	1(6)	-	-	-	-

**5q del**	1(6)	-	4(67)	-	-

**11;23**	1(6)	-	-	-	-

## References

[1] Döhner H., Estey E. H., Amadori S. (2010). Diagnosis and management of acute myeloid leukemia in adults: recommendations from an international expert panel, on behalf of the European LeukemiaNet. *Blood*.

[2] Mrózek K. (2008). Cytogenetic, Molecular Genetic, and Clinical Characteristics of Acute Myeloid Leukemia With a Complex Karyotype. *Seminars in Oncology*.

[3] Mrózek K., Marcucci G., Nicolet D. (2012). Prognostic Significance of the European LeukemiaNet Standardized System for Reporting Cytogenetic and Molecular Alterations in Adults With Acute Myeloid Leukemia. *Journal of Clinical Oncology*.

[4] Röllig C., Bornhäuser M., Thiede C. (2011). Long-Term Prognosis of Acute Myeloid Leukemia According to the New Genetic Risk Classification of the European LeukemiaNet Recommendations: Evaluation of the Proposed Reporting System. *Journal of Clinical Oncology*.

[5] Faguet B. G. (2001). *Hematologic Malignancies, Methods and Techniques*.

[6] Yaghmaie M., Gerayeli N., Ghaffari S. H., Ghavamzadeh A., Tootian S. M. (2009). Some specific chromosomal aberrations of hematologic malignancies in 80 Iranian population. *International Journal of Hematology-Oncology and Stem Cell Research*.

[7] Seabright M. (1971). A rapid banding technique for human chromosomes. *The Lancet*.

[8] Grimwade D., Walker H., Harrison G. (2001). The predictive value of hierarchical cytogenetic classification in older adults with acute myeloid leukemia (AML): Analysis of 1065 patients entered into the United Kingdom Medical Research Council AML11 trial. *Blood*.

[9] Fenaux P., Preudhomme C., Laï J. L., Morel P., Beuscart R., Bauters F. (1989). Cytogenetics and their prognostic value in de novo acute myeloid leukaemia: a report on 283 cases. *British Journal of Haematology*.

[10] Grimwade D., Walker H., Oliver F. (1998). The importance of diagnostic cytogenetics on outcome in AML: analysis of 1,612 patients entered into the MRC AML 10 trial. The Medical Research Council Adult and Children's Leukaemia Working Parties. *Blood*.

[11] Yunis J. J., Lobell M., Arnesen M. A. (1988). Refined chromosome study helps define prognostic subgroups in most patients with primary myelodysplastic syndrome and acute myelogenous leukaemia. *British Journal of Haematology*.

[12] Hda N., Chadli B., Bousfiha A., Trachli A., Harif M., Benslimane A. (1996). Cytogenetic survey of 53 Moroccan patients with acute myeloblastic leukemia. *Cancer Genetics and Cytogenetics*.

[13] Stölzel F., Mohr B., Kramer M. (2016). Karyotype complexity and prognosis in acute myeloid leukemia. *Blood Cancer Journal*.

[14] Harani M. S., Adil S. N., Kakepeto G. N., Khijli Z., ShaikhMU Khurshid M. (2006). Significance of cytogenetic abnormalities in acute myeloid leukemia. *Journal of Pakistan Medical Association*.

[15] Zehra S., Najam R., Farzana T., Shamsi T. S. (2016). Outcomes of 1st remission induction chemotherapy in acute myeloid leukemia cytogenetic risk groups. *Asian Pacific Journal of Cancer Prevention*.

[16] Luquet I., Laï J. L., Barin C. (2008). Hyperdiploid karyotypes in acute myeloid leukemia define a novel entity: A study of 38 patients from the Groupe Francophone de Cytogenetique Hematologique (GFCH). *Leukemia*.

[17] Yunis J. J., Rydell R. E., Oken M. M., Arnesen M. A., Mayer M. G., Lobell M. (1986). Refined chromosome analysis as an independent prognostic indicator in de novo myelodysplastic syndrome. *Blood*.

[18] Haase D., Germing U., Schanz J. (2007). New insights into the prognostic impact of the karyotype in MDS and correlation with subtypes: evidence from a core dataset of 2124 patients. *Blood*.

[19] Yang X., Sun A., Yin J. (2012). Monosomal Karyotypes among 1147 Chinese Patients with Acute Myeloid Leukemia: Prevalence, Features and Prognostic Impact. *Asian Pacific Journal of Cancer Prevention*.

[20] Cortes J. E., Kantarjian H., O'Brien S. (1995). Clinical and prognostic significance of trisomy 21 in adult patients with acute myelogenous leukemia and myelodysplastic syndromes. *Leukemia*.

[21] Coniat M. B.-L., Khac F. N., Daniel M.-T., Bernard O. A., Berger R. (2001). Chromosome 21 abnormalities with AML1 amplification in acute lymphoblastic leukemia. *Genes, Chromosomes and Cancer*.

[22] Volkert S., Kohlmann A., Schnittger S., Kern W., Haferlach T., Haferlach C. (2014). Association of the type of 5q loss with complex karyotype, clonal evolution, TP53 mutation status, and prognosis in acute myeloid leukemia and myelodysplastic syndrome. *Genes, Chromosomes Cancer*.

[23] Giagounidis A. A. N., Germing U., Strupp C., Hildebrandt B., Heinsch M., Aul C. (2005). Prognosis of patients with del(5q) MDS and complex karyotype and the possible role of lenalidomide in this patient subgroup. *Annals of Hematology*.

[24] Xu L., Zhao W., Xiong S. Molecular cytogenetic characterization and clinical relevance of additional, complex and/or variant chromosome abnormalities in acute promyelocytic leukemia. *Leukemia*.

[25] Sgherza N., Abruzzese E., Perla G. (2017). Onset of chronic myeloid leukemia with complex karyotype in a pregnant patient: Case report and revision of literature. *Therapeutics and Clinical Risk Management*.

[26] Yi-Kong Keung., Mark J., Julia M., Bayard L., Ralph D., David H. (2001). Bone Marrow Cytogenetic Abnormalities of Aplastic Anemia. *American Journal of Hematology*.

[27] Kim S. Y., Kim H. J., Kang H. J. (2013). Clinical significance of cytogenetic aberrations in bone marrow of patients with diffuse large B-cell lymphoma: Prognostic significance and relevance to histologic involvement. *Journal of Hematology & Oncology*.

